# Yellow Fever

**Published:** 1886-05

**Authors:** 


### YELLOW FEVER.

There seems to be no doubt that a discovery has been made which will prove one of the most important in the age of discoveries and check the ravages of yellow fever or prevent its effects entirely. That it has proved successful there seems to be no room for doubt. We copy the letter of Dr. Gaston of Atlanta, Ga., and that of Dr. Dr. Freire, of Rio Janeiro, on this very important subject:

107 Marietta St., Atlanta, Ga.,
January 30, 1886.

Dear Dr. Vander Veer:

Yours of the 22d inst. came duly to hand. * * * * I would again draw your attention to the subject of yellow fever inoculation, as noticed in the numbers of *Gaillard's Journal* for December, 1885, and January, 1886, by which you will see that I have taken an active part in urging forward this prophylactic measure. Another letter has just reached me from Dr. Freire, of which I enclose you a translation, written by one of my daughters, who was born in Brazil during our sojourn in that empire. * * *

Very truly yours,

I. M. F. Gaston.

The following is the translation referred to by Dr. Gaston:

Rio de Janeiro, December 27, 1885.

Dear Dr. Gaston:

It is with great satisfaction that I reply to your kind letter just received, to which I hasten my answer.

The lively interest which you have shown in presenting my discovery to the public is so gratifying to me that I cannot find words which express adequately my deep sense of obligation. I have entire confidence that, under the protection of your important name, my discovery of the prevention of yellow fever by means of inoculation with attenuated cultures will be shortly extended throughout the grand American Republic heretofore devasted by that scourge.

The day on which this happens will be that of the richest reward for the great labors and hard sacrifices which I have undertaken in the fulfillment of this humane problem, and your name will be found linked with this work of Christian charity and progressive civilization.

I have read with all attention your correspondence, as likewise the authoritative opinion of Dr. Holt, to whom I write at present date. His ideas will most assuredly be duly weighed by the American Association of Public Health, and I judge that the commission of medical men, of which you spoke, will come very soon to give us the distinguished honor of verifying the results of our prophylactic measure, which up to this time has been attended with such notable success that not one of the six thousand who were inoculated during the present year has died of yellow fever, while nearly five hundred who were not inoculated have sunk into the grave as victims of that disease.

In a short time I will send you a pamphlet in which I give minute information upon the inoculations practiced, by which all may properly estimate the confidence which ought to be reposed in the preventive method that I propose.

I am, dear sir, with profound respect, your obedient servant,

DOMINGOS FREIRE,

No. 29 Cattete Street.

